# Severe heterotopic ossification after total hip arthroplasty in male patients under 70 years of age: effectiveness of prophylactic protocol

**DOI:** 10.1007/s12306-024-00868-4

**Published:** 2024-10-09

**Authors:** Alessandro Aprato, Simone Cambursano, Stefano Artiaco, Federico Fusini, Simone Bevilacqua, Paolo Catalani, Alessandro Massè

**Affiliations:** University of Turin, Corso Dogliotti, 14, 10100 Turin, Italy

**Keywords:** Total hip arthroplasty, Heterotopic ossification, Elderly

## Abstract

**Background:**

This study aims to evaluate the incidence of clinically significant heterotopic ossification (HO) in primary total hip arthroplasty (THA), comparing outcomes with and without the adoption of an HO prophylactic protocol in male patients under 70 years of age.

**Methods:**

The prophylactic protocol involved the administration of 50 mg of Indomethacin twice daily for 3 weeks. HO presence was classified according to the Brooker classification system, considering “severe” clinically significant HO (Brooker grade 3 and 4).

**Results:**

Two hundred and seventy-nine patients were included in our study, and an overall HO rate of 68.2% versus a rate of 61.5% was found respectively in patients not subjected and subjected to prophylactic protocol, without significant difference (PR 0.062). However, patients not subjected to the HO prophylactic protocol exhibited a severe HO rate of 22.4% compared to 7.7% in the prophylactic group, with a statistically significant difference (*P* = 0.008).

**Conclusions:**

Our study demonstrated that prophylactic protocol adoption is significantly associated with lower rate of severe HO in male patients under 70 years of age. Currently, there are no orthopedic guidelines for the prevention and management of HO after THA, but in the absence of contraindications, the adoption of a prophylactic protocol for HO should always be considered in high-risk patients.

## Introduction

Total hip arthroplasty (THA) has a worldwide increasing diffusion [1, 2], showing good long-term results [3]. One common complication of this procedure is heterotopic ossification (HO) [4]: an abnormal formation of bone in extra-skeletal soft tissue [5, 6]. HO can lead to significant functional limitations and decreased satisfaction rates [7–9]. Zhu al [10] reported an average HO rate of 30% after THA, while other studies have documented rates ranging from 15 and 90% [11, 12]. Conversely, a recent meta-analysis demonstrated a 54–64% reduction in HO incidence with an adequate prophylactic protocol [13].

The most common combination of risk factors is male gender and young age [10, 14] representing an active and highly functional demographic where clinically severe HO could be a significant complication that should be avoided with adequate prophylactic treatment.

The aim of this study is to compare the incidence of clinically significant HO in two groups of male patients under 70 years of age following THAs performed with a posterolateral approach; the control group did not receive a prophylactic protocol, while the study group received 50 mg of Indomethacin twice daily for 3 weeks beginning the day after surgery.

## Materials and methods

This retrospective study was conducted at a single large teaching hospital, with the study protocol approved by the local committee (2018/2022), trial number 038.815 (final approval on 26/6/2019). Male patients under 70 years of age who underwent a THA with a posterolateral approach during a 15-year period were included. Patients were assigned to the control group if they did not receive the HO prophylactic protocol, or to the study group if they received 50 mg of Indomethacin twice daily for 3 weeks starting the day after surgery.

Exclusion criteria included the absence of X-ray follow-up longer than 2 years or simultaneous bilateral THA.

Data collected for both groups included age at surgery, preoperative diagnosis (primary or secondary hip arthritis, femoral or acetabular fracture), and the presence of relevant systemic diseases.

Two orthopedic surgeons reviewed the 2-year follow-up X-rays and classified the HO (if present) according to the Brooker classification system [15]: no ossification (grade 0), islands of bone in the soft tissues around the hip (grade 1), bone spurs from the proximal femur or pelvis with at least 1 cm between opposing bone surfaces (grade 2), bone spurs from the proximal femur or pelvis with less than 1 cm between opposing bone surfaces (grade 3) and apparent bone ankyloses of the hip (grade 4). According to literature [10, 11, 16], we considered “severe” clinically significant HO (Brooker grade 3 and 4).

### Statistical analysis

All data were analyzed with standard descriptive statistics. T test and Chi^2^ test were employed to compare the two groups in terms of age, preoperative diagnosis, and presence systemic diseases.

Univariate analysis with the Chi^2^ test was performed regarding severe HO presence (yes or no).

The relationship between severe HO and patients’ characteristics was assessed with multivariate linear regression models. *P* values lower than 0.05 were considered statistically significant. All analyses were performed using Stata version 12 (Stata Corporation, College Station, TX, USA).

## Results

During the study period, 362 male patients under 70 years of age underwent a THA with posterolateral approach. In 75 cases, no X-ray follow-up longer than 2 years was retrievable, and eight patients underwent a simultaneous bilateral THA; therefore, 279 patients were included and assigned to the two groups; control group was composed by 214 patients, while remaining were included in the study group. The mean age was 52 and 52.7 years old respectively for the first and the second group, without a statistically significant difference (Pr = 0.64).

The preoperative diagnosis was primary arthritis in 53.1% of study group and 47.7% of control group, respectively, in 44.1% and 47.2% diagnosis was secondary arthritis, and post-traumatic in 3% and 7%. The two groups were not significantly different in terms of preoperative diagnosis (Pr = 0.277).

At the time of surgery, 20.3% of patients presented systemic diseases in the study group and 16.4% in control group. The two groups were not significantly different in terms of systemic diseases (Pr 0.063).

HO was found in 66.7% of cases; in the control group, the overall rate found was 68.2%, while in the case group, it was 61.5% with no statistically significant differences (PR 0.062). According to the Brooker classification system, 35.5% of patients were classified as grade 1, 12.2% as grade 2, 10.8% as grade 3 and 8.2% as grade 4 (Table 1).

**Table 1:** Patients classified according to Brooker classification system, with and without adoption of HO prophylactic protocol

	Brooker 0	Brooker 1	Brooker 2	Brooker 3	Brooker 4	Total
Control group	68	70	28	27	21	214
Study group	25	29	6	3	2	65
Total	93	99	34	30	23	279

Analyzing only the severe HO, control group showed severe HO rate of 22.4% versus 7.7% found in the study group (*P* = 0.008).

HO prophylactic protocol adoption was found to be significantly and independently associated with lower rate of severe HO in the multivariable analysis (Table 2).

**Table 2:** Multivariable analysis showing statistically significant association between HO prophylactic protocol adoption and lower rate of severe HO

	Coefficient	Std. Error	*P*	95% Conf. Interval
Age	0.007	0.017	0.665	− 0.025/0.04
Preop diagnosis	− 0.312	0.288	0.279	− 0.877/0.253
General diseases	− 0.147	0.417	0.724	− 0.96/0.67
Prophylaxis protocol	− 1.303	0.497	0.009	− 2.27/− 0.33

## Discussion

Currently, there are no orthopedic guidelines about prevention and management of HO after THA [12, 17]. To date, for HO prevention, radiotherapy has the best prophylaxis effectiveness for HO after THA but presents higher cost and complex management: for these reasons may be the most appropriate approach in high-risk patients (ipsilateral high-grade HO) or in those with contraindications to NSAIDS [17–20].

Nowadays, NSAIDs are the most frequently utilized prophylactic agents [17, 21]. Specifically, selective cyclooxygenase 2 (COX-2) inhibitors have demonstrated comparable effectiveness in preventing HO formation following hip surgery, while exhibiting reduced gastrointestinal side effects compared to non-selective NSAIDs [18, 19].

To date, based on our knowledge, there are no other scientific studies focusing specifically on HO after THA in high-risk patients such as males under 70 receiving Indomethacin prophylaxis. Therefore, direct comparison of our results with others is challenging. However, some findings in the literature show comparable values to ours. For instance, in the study by Barbato et al. [22], patients without prophylaxis exhibited an HO incidence of 55% and 8.9% when considering only Brooker 3 and 4 cases. Conversely, in our study, patients undergoing indomethacin prophylaxis did not experience any cases of Brooker 3 or 4 HO, with an overall HO incidence of 23.1%. It is important to note that these studies involved a less standardized patient population, and their prophylaxis regimen was based on celecoxib.

In our study, the overall incidence of HO was 66.7% of cases, similar to rates reported by other authors, which can be as high as 90% [12, 23]. According to the literature, approximately 15% of patients with HO following THA experience pain and restricted range of motion in the affected hip [8].

Our study focused on a cohort of male patients under 70 years of age, a demographic we identified as being at highest risk. Male sex is widely acknowledged as a significant risk factor for clinically relevant HO following THA [10, 24, 25]. Regarding age, there is not complete consensus among researchers: Zhu et al. [10] and Eggli et al. [24] argue that age is not a risk factor for HO in THA, while Biz [26] suggests a correlation between younger age and HO formation.

Eggli et al. [7] showed that patient satisfaction after a THA was significantly influenced by HO degree, dropping from almost 90% in Brooker grade 0 to less than 30% in Brooker grade 3 and 4. Also patients’ rate with mild or severe pain was influenced by HO, increasing from less than 10% to more than 50% from Booker grade 0 to Brooker grade III and IV. Moreover, it is reported that walking capacity decrease and use of analgesic increase statistically in the highest grades of Brooker classification [7].

According to our experience, according to these last considerations and to other authors [10, 11, 16], we considered only “severe” clinically significant HO (Brooker grade 3 and 4).

Male patients under 70 years of age are usually the most demanding patients in which the presence of HO, in particular high-grade HO, could be more disabling: for these reasons, we choose this cohort and this cut-off on Brooker grade.

As we already said, there is still not a complete concordance between authors [10, 24, 26] if age is or not a risk factor for HO formation; in any case in our study, there is no age difference between groups subjected and not subjected to HO-prophylaxis protocol. Some authors showed that a diagnosis of primary or secondary arthritis could be a risk factor for HO respect a femoral neck fracture [26], such as the presence of systemic diseases respect its absence [10, 27]; in our study, there are no difference about these risk factors between groups of patients subjected or not to prophylactic protocol.

According to Oni et al. [16], our study showed that the prophylaxis protocol has the most effectiveness in prevention of severe HO, the most disabling HO form in particular in our cohort of patients and highlighted as prophylactic protocol adoption was independently associated with lower rate of severe HO.

This study shows that taking a postoperative protocol with Indomethacin can statistically significantly reduce the percentage of severe HO (Brooker 3 and 4). The fact that the same statistically significant difference was not identified for mild HO can be explained by two reasons: firstly, this is a high-risk subpopulation in which the formation of calcifications cannot be reduced to zero to date, and secondly, the prophylaxis implemented may have reduced the natural pathophysiological process of heterotopic calcification so that they would stop at milder forms instead of progressing to more severe stages.

Our study presents several limits. Its retrospective single-center nature may limit our conclusions. Furthermore, we used the Brooker classification system; based on a single anteroposterior X-ray of the pelvis, it may under- or overestimate the HO extension but it is still the most widely diffused classification system 9, 28. Eventually, clinical evaluation was not performed. Therefore, further clinical study should confirm our statements.

## Conclusions

In patients at high risk of developing heterotopic ossification (HO) following THA, prophylaxis protocol with Indomethacin effectively reduces the incidence of severe HO. Therefore, its adoption should always be considered where not contraindicated. The persistence of low-grade HO suggests that Indomethacin could primarily control the progression rather than the initial formation of calcifications. Further, prospective, randomized trials and multicenter studies are necessary to fully elucidate their pathogenesis and to develop optimized prophylactic protocols.

## Data Availability

All data have been stored in the dedicated repository of University of Turin.
